# Pulmonary Function in Infants with Swallowing Dysfunction

**DOI:** 10.1371/journal.pone.0123125

**Published:** 2015-05-15

**Authors:** James D. Tutor, Saumini Srinivasan, Memorie M. Gosa, Thomas Spentzas, Dennis C. Stokes

**Affiliations:** 1 Program in Pediatric Pulmonary Medicine, University of Tennessee Health Science Center, Le Bonheur Children’s Hospital and St. Jude Children’s Research Hospital, Memphis, TN, United States of America; 2 Rehabilitation Services—Department of Speech-Language Pathology, Le Bonheur Children’s Hospital, Memphis, TN, United States of America; 3 Departments of Pediatrics and Preventive Medicine, University of Tennessee Health Science Center, Le Bonheur Children’s Hospital, Memphis, TN, United States of America; 4 Department of Communicative Disorders, The University of Alabama, Tuscaloosa, AL, United States of America; The Ohio State Unversity, UNITED STATES

## Abstract

**Background:**

Swallowing dysfunction can lead to recurring aspiration and is frequently associated with chronic symptoms such as cough and wheezing in infants. Our objective was to describe the characteristics of infants with swallowing dysfunction, determine if pulmonary function abnormalities are detectable, and if they improve after therapy.

**Methods:**

We studied 38 infants with a history of coughing and wheezing who had pulmonary function tests performed within two weeks of their diagnosis of swallowing dysfunction. The raised lung volume rapid thoracoabdominal compression technique was used. After 6 months of therapy, 17 of the infants repeated the tests.

**Results:**

Initially, 25 had abnormal spirometry, 18 had abnormal plethysmography, and 15 demonstrated bronchodilator responsiveness. Six months later test were repeated for seventeen patients. Ten patients had continued abnormal spirometry, two patients remained normal, three patients’ abnormal spirometry had normalized, and two patients’ previously normal studies became abnormal. Eight of the 17 patients had continued abnormal plethysmography, six had continued normal plethysmography, and three patients’ normal plethysmography became abnormal. After 6 months of treatment, eight patients demonstrated bronchodilator responsiveness, of which five continued to demonstrate bronchodilator responsiveness and three developed responsiveness. The remainder either continued to be non- bronchodilator responsive (two) or lost responsiveness (three.) The findings of the abnormal tests in most infants tested is complicated by frequent occurrence of other co-morbidities in this population, including gastroesophageal reflux in 23 and passive smoke exposure in 13 of the infants.

**Conclusions:**

The interpretation of lung function changes is complicated by the frequent association of swallowing dysfunction with gastroesophageal reflux and passive smoke exposure in this population. Six months of medical therapy for swallowing dysfunction/gastroesophageal reflux did not significantly improve pulmonary function in these infants. Long-term studies will be necessary to determine which of these changes persists into adulthood.

## Introduction

Aspiration, the inhalation of foreign material into the lower airway, has been a significant cause of morbidity and mortality throughout history. Aspiration can be an acute event or a chronic recurrent syndrome. It may occur with swallowing during oral feeding or after feeding during episodes of gastroesophageal reflux (GER), the retrograde movement of gastric contents across the lower esophageal sphincter into the esophagus [1].

Aspiration may occur in children who have problems with dysphagia, difficult or improper swallowing of liquids, solids, or even saliva. When aspiration is chronic and recurrent, the effects on lung development can be devastating, leading to pulmonary problems such as recurrent wheezing, recurrent pneumonias, and the development of severe impairment of lung function and pulmonary scarring that can occasionally lead to death. Though the exact incidence of dysphagia in children and accompanying aspiration is unknown, it is felt to be significant and it is frequently unrecognized by primary care physicians or caregivers as a cause of chronic respiratory symptoms [2].

Pulmonary function tests (PFTs), to measure lung function in infants and help evaluate the severity of pulmonary disease, became available in the 1980s [3]. Initial infant PFTs measured maximal expiratory flow at functional residual capacity (V_maxFRC_) after rapid thoracoabdominal compression (RTC), with an intra-subject variability of 11%-36% [4]. In the mid-1990s, the technique of RTC from lung volumes near total lung capacity was described with an intra-subject variability of < 5% [5]. Normative data for FEFs (forced expiratory flow), lung volumes and bronchodilator responsiveness were published in 2001 [6,7] using equipment and methods described by Feher *et al*. in 1996 [8]. Guidelines for performance of raised volume forced expirations [9] and for measurement of infant lung volumes by body plethysmography [10] in infants are now available.

PFTs in infants with respiratory symptoms and gastroesophageal reflux (GER) have been reported [11–13]. However, no studies of PFTs in infants with respiratory symptoms and diagnosed with swallowing dysfunction have been reported so far. This prospective observational pilot study documents PFT results in a group of infants diagnosed with swallowing dysfunction, many of whom also had concomitant GER.

## Materials and Methods

This study was conducted in accordance with the amended Declaration of Helsinki and approved by the Institutional Review Board at the University of Tennessee Health Science Center in Memphis (protocol # 08-08819-FB). Parents/guardians of study participants provided written informed consent.

### Study subjects

Infants with a history of respiratory symptoms, such as coughing, wheezing, or recurrent pneumonias, and referred to Le Bonheur Children’s Hospital from July 1, 2008 through June 30, 2011 were initially evaluated for possible recruitment into this study. Of those infants, only neurologically normal infants born at term, between the ages of 1–24 months, weighing between 5–15 kg and < 90 cm tall, with swallowing dysfunction newly diagnosed by videofluoroscopic swallowing study (VFS) were eligible to participate in the study. Exclusion criteria were: a previous diagnosis of sleep apnea, bronchopulmonary dysplasia, cystic fibrosis, immunodeficiency upper airway obstruction, neuromuscular or central nervous system disease, craniofacial abnormalities, Arnold-Chiari malformation, tracheoesophageal fistula, vascular ring, pharyngeal and laryngeal anomalies, laryngotracheal cleft, velopalatal insufficiency, seizure disorder, or unstable cardiac, hepatic, or renal disease, or a history of an adverse reaction to chloral hydrate. Also, infants with an acute respiratory tract infection within one month of being screened for the study were excluded.

### Videofluoroscopic swallowing study protocol

VFS studies were performed using a standard clinical protocol [14,15]. Testing was performed by a speech language pathologist and radiologist with the infant seated semi-upright and using fluoroscopy of the upper airway in the lateral projection. A prepackaged liquid with standard viscosity, Varibar Thin Liquid Barium (target viscosity 4 centipoise, range < 15), was fed to the infant from a Similac disposable bottle with a standard one-hole nipple. Depending on the infant’s observed swallowing function, compensatory thickened liquids—first thickened to the nectar (target viscosity 300 centipoise, range 150–450), then honey (target viscosity 1500 centipoise, range 800–1800) consistencies—were also fed to the infant, if necessary, with either a standard one-hole nipple, cross-cut or red fast-flow nipple, and fluoroscopy was repeated during feeding [14–16]. The penetration-aspiration score [17] (PAS) was used to provide an objective rating of laryngeal penetration and observed aspiration events. For the purposes of this study, a PAS of 1 indicated no airway compromise, 2 was classified as airway compromise of mild severity, 3–5 as moderate airway compromise, and 6–8 as severe airway compromise. Each subject was classified as having mild, moderate, or severe airway compromise based on ratings from the PAS. The diagnosis of swallowing dysfunction was made by review of the complete MBS and consensus of speech-language pathologist and radiologist as to classification of mild, moderate, or severe swallowing dysfunction. The same speech-language pathologist provided the PAS ratings for all of the infants in the study and was blinded to PFT results.

### Study protocol

The initial PFT was performed within two weeks of the diagnosis of swallowing dysfunction and the second approximately 6 months later. Between the tests, each subject received appropriate medical or surgical therapy for swallowing dysfunction, and, if also present, GER. Prior to each set of tests, the principal investigator reviewed birth history, gastrointestinal symptoms, respiratory symptoms, feeding history, history of daycare and secondhand smoke exposure (both assessed by history from the caregiver), allergies, relevant past medical history and hospitalizations/surgeries, family history of gastrointestinal or respiratory diseases, and medical/pharmacological/surgical therapies received for treatment of swallowing dysfunction or GER. The principal investigator reviewed each subject’s available respiratory and gastrointestinal radiological studies, and performed a brief examination with particular emphasis paid to respiratory tract findings.

#### Protocol for performance of infant PFTs

All infant PFTs were performed at Le Bonheur Children’s Hospital utilizing the Infant Pulmonary Laboratory (Collins, Inc., Braintree, MA). Prior to each test, each subject was fasted according to the sedation policy utilized at our institution, and sleep deprived by the parent/guardian for 3–4 hours prior to the test. Each subject received (by inserted nasogastric tube or by gastrostomy tube, if one was present) chloral hydrate 75–100 mg/kg. The nasogastric tube was removed once the subject was adequately sedated.

PFTs consisted of measurement of pre- and post-bronchodilator spirometry utilizing RTC from lung volumes at total lung capacity [9]. Lung volumes were measured using body plethysmography [10]. The FEFs were initiated after inflation of each subject’s lungs to an airway pressure of 30 cm water and continued to residual volume. The RTC procedure began with a jacket rise time of < 100 msec starting at a pressure of 50 cm water. The jacket pressure was increased incrementally with each successive maneuver until a pressure was reached that produced no further increase in airflow. The maximum pressure that could be applied during the test was 110 cm water. Maneuvers were repeated until at least three technically satisfactory and reproducible flow-volume curves were recorded.

Values measured were forced vital capacity (FVC), forced expired volume at 0.5 second (FEV_0.5_), FEV_0.5_/FVC, forced expiratory flows (FEFs) at 25%, 50%, 75%, and 85% of expired FVC (FEF_25%_, FEF_50%_, FEF_75%_, FEF_85%_), and FEF between 25% and 75% of expired FVC (FEF_25%-75%_). Test values were reported from the best flow-volume curve (i.e., that with the highest product of FVC and either FEV_0.5_ or FEF_0.75_). Lung volume values reported were total lung capacity (TLC), functional residual capacity (FRC), expiratory reserve volume (ERV), residual volume (RV), RV/TLC, and FRC/TLC. Test values were reported as percentage of predicted values and as Z-scores. Results were interpreted using normative data for FEFs in infants [7].

For bronchodilator responsiveness testing, each subject received albuterol using a metered-dose inhaler with a spacer in a dose of six puffs. Each puff was followed by an inflation of the lungs to 30 cm H_2_O. The RTC was repeated approximately 20–30 minutes after the last puff of albuterol had been given. Bronchodilator responsiveness, expressed as percent change from the baseline values, was assessed using normative data from normal infants.^6^

### Statistical analysis

Statistical analysis was performed using IBM SPSS 20. Results were expressed as median and interquartile range 25% to 75% and normally distributed as mean ± standard deviation (SD). The association of the interpretations of the initial and 6-month PFTs was tested with McNemar’s test.^18^ Values from the initial and 6-month PFTs were compared using the Wilcoxon signed rank test.^19^ Test results were considered statistically significant at a *p* value of less than 5% (*p* < 0.05).

## Results

### Subject characteristics

Thirty-eight infants newly diagnosed with swallowing dysfunction by VFS who met study inclusion criteria were enrolled. The mean age ± SD at the time of the first tests was 12.6 ± 5.19 months, and at the second test it was 18.7 ± 5.33 months. The number and percentage of infants with symptoms, a history of clinical feeding difficulties (choking, gagging, and vomiting), passive tobacco smoke exposure in the home (defined as one or both caregivers smoking), and exposure to daycare are shown in Table 1. GER was diagnosed based on barium esophagogram or gastric scintiscan results; seven of the 38 were not tested, and the test was negative in eight of the 38 infants. Sixteen of 33 infants who had chest radiographs performed prior to the diagnosis of swallowing dysfunction showed abnormalities (peribronchial thickening, hyperinflation, atelectasis, pneumonia). Of the 38 infants, 20 were classified as having mild airway compromise, 15 were classified as having moderate airway compromise, and three were classified as having severe airway compromise.

**Table 1:** Clinical characteristics of study subjects (n = 38).

Age at presentation (months)	12.6 ± 5.19
Race	19 (50%) African American, 19 (50%) Caucasian
Gender	12 (32%) female, 26 (68%) male
Cough/wheezing	37 (97%)
Gastroesophageal reflux (documented)	23 (60%)
Symptoms of swallowing dysfunction	26 (68%)
Passive smoke exposure	13 (34%)
Daycare exposure	18 (47%)
Family history of asthma/atopy	34 (90%)
Abnormal chest X-ray	16/33 (48%)

All 38 subjects performed PFTs within two weeks after the diagnosis of their swallowing dysfunction. Despite multiple attempts to contact the parents of the study subjects by telephone or mail, only 17 of the 38 infants returned for a second PFT about six months after being prescribed therapies for their swallowing dysfunction (changes in positioning, changes/modifications to utensils including bottle/nipple systems, and modifications to the viscosity of liquids by thickening them), and, if present, for GER (use of inhibitors of histamine H_2_ receptors or proton pump inhibitors). Demographics for the 17 patients are displayed in Table 2. Most patients (14/17) reported compliance with medical and/or feeding therapies.

**Table 2:** Demographics of patients who had both first and second pulmonary function tests.

Subject	Gender/race	Cough/wheeze	CXR abnormality	PA score	GER	Smoke exposure
1	M/AA	Y	Atelectasis	6	NE	N
2	F/AA	Y	None	2	Y	N
3	M/C	Y	None	2	Y	N
4	M/C	Y	None	2	N	N
5	F/AA	Y	None	4	Y	Y
6	M/C	Y	None	2	Y	Y
7	M/AA	Y	None	2	NE	N
8	M/C	Y	Atelectasis, PBT	2	Y	N
9	F/AA	Y	None	2	Y	Y
10	F/C	Y	Hyperinflation, PBT	2	Y	Y
11	M/C	Y	None	4	Y	N
12	F/AA	Y	None	4	Y	Y
13	M/AA	Y	None	2	NE	N
14	M/C	Y	None	4	N	Y
15	M/AA	Y	Not done	1	N	Y
16	M/C	Y	Pneumonia	2	Y	N
17	M/C	Y	Atelectasis	2	Y	Y

M, male; F, female; AA, African American; C, Caucasian; Y, yes; N, no; NE, not evaluated; PA, penetration-aspiration, PBT, peribronchial thickening.

### Pulmonary function test results

**Fig 1:**
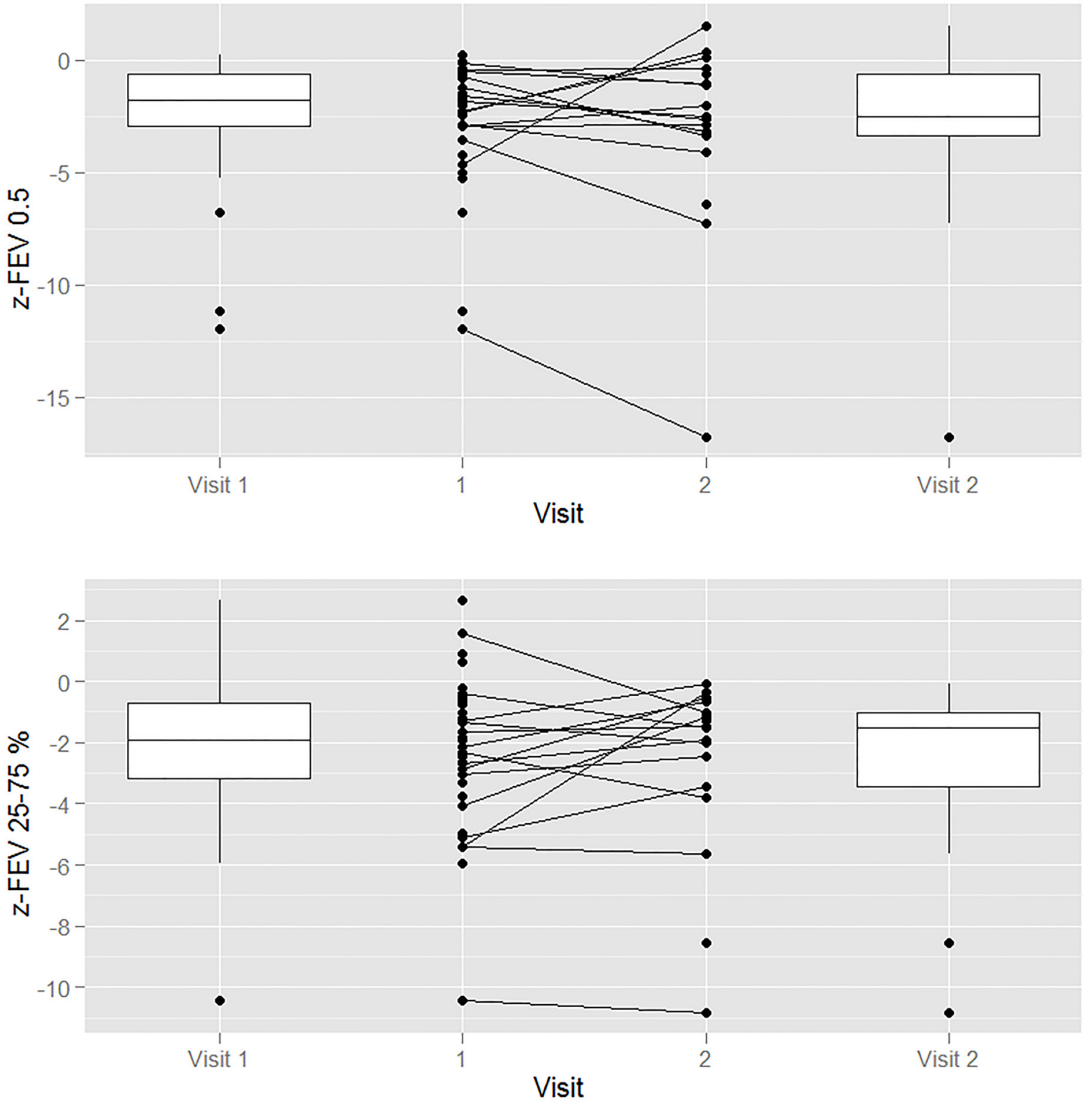
The FEV_0.5_ and FEF_25–75%_ Z-scores for the first and second pulmonary function tests as boxplots of the median and interquartile ranges with lines between indicating the individual changes. FEF, forced expiratory flow; FEV, forced expiratory volume.

Thirty-eight patients had spirometry at the first visit, and 25 (66%) had abnormal results (decreased FEV_0.5_ and FEF_25–75%_ percent of predicted and Z-scores). Fig 1 presents the FEV_0.5_ and FEF_25–75%_ Z-scores for the patients for the first and second PFTs as boxplots of the median and interquartile ranges with lines between indicating the individual changes. Seventeen of the 38 patients had repeat spirometry six months later. Tables 3 and 4 display the results of the first and second PFTs, respectively. Of the 17, 10 had abnormal spirometry on both the initial and follow-up tests, two had normal tests at both visits, three had abnormal initial tests that normalized, and two had normal tests that became abnormal. All three patients who had abnormal spirometry and subsequently normalized were compliant with their treatment.

**Table 3:** Initial pulmonary function test results for subjects who underwent repeat testing 6 months after therapy.

Subject	Age (weeks)	Height (cm)	FEV_0.5_ Z-score	FEF_25–75%_ Z-score	TLC % pre	RV % pre	FEV_0.5_% change after BD	FEF_25–75%_ % change after BD
1	35.3	67.2	-0.5	1.58	100	160	-2.3	1.1
2	28.9	62.5	-0.16	-1.31	106	77	9.7	25.3
3	80.9	80	-4.64	-5.42	94	ND	-19.1	-21.8
4	27.1	64.5	-2.96	-5.09	106	139	11.2	17.3
5	33.7	63	-2.31	-4.08	95	107	26.1	71.7
6	34.1	57	-0.47	-2.16	103	82	4.4	-0.9
7	29	61	-3.57	-5.42	86	94	14.4	43
8	14.9	53	-0.48	-3.88	120	94	ND	ND
9	55.4	73	-1.86	-1.35	65	59	3.5	4.4
10	32	64.5	-1.25	-2.32	89	90	23.7	20.7
11	58.7	75	-0.76	-0.41	94	125	-8.7	-6.6
12	43.9	68	-2.19	-3.12	85	91	2.9	-2.2
13	43.7	70.5	-11.95	-10.45	ND	ND	0	77.8
14	67.1	74	-2.97	-3.05	78	86	35.7	52.9
15	51.7	76	-2.29	-2.88	82	97	17.6	61.7
16	73	80	-1.6	-1.64	102	155	9.5	39.8
17	67.7	81.5	-2.87	-2.7	71	101	-16.5	-10.9

BD, bronchodilator; FEF_25–75%_, forced expiratory flow between 25% and 75% of expiration; FEV_0.5_, forced expiratory volume during the first half-second of expiration; ND, not done; RV, residual volume; TLC, total lung capacity; % pre, percent predicted.

**Table 4:** Repeat pulmonary function test results for subjects who underwent repeat testing 6 months after therapy.

Subject	Age (weeks)	Height (cm)	FEV _0.5_ Z-score	FEF_25%75%_ Z-score	TLC % pre	RV % pre	FEV _0.5_ % change	FEF_25%75%_ % change
1	61	83.5	-1.07	-1.04	154	ND	ND	ND
2	54.4	79.5	-1.12	-0.09	80	83	10.2	8.1
3	105.9	91	1.53	-0.36	72	67	10.2	22
4	60	81.5	-2.87	-3.42	96	127	36.7	53.2
5	60.7	69	0.36	-1.16	103	93	2.6	24.8
6	51.6	76	-0.38	-0.67	88	83	10.1	31.6
7	56	73.5	-7.29	-5.65	66	151	ND	ND
8	40.7	66.5	-0.61	-1.31	92	91	-4.8	-10.5
9	82.6	82	-2.55	-2.01	77	96	-2.8	-9.1
10	63.9	76.5	-3.19	-3.78	80	132	1.9	8.3
11	58.7	75	-3.4	-1.51	90	67	72.7	46.5
12	70	75	-6.44	-8.53	ND	ND	57.7	108.4
13	83	81.4	-16.79	-10.82	54	118	-39.4	12.8
14	109.1	84	-2.04	-2.45	76	96	ND	ND
15	82.7	82.5	-0.12	-0.53	95	87	5.3	28.3
16	98	87	-2.66	-1.47	73	112	-2.7	-12.1
17	107.7	88	-4.11	-1.9	61	109	-5.8	-26.4

BD, bronchodilator; FEF_25–75%_, forced expiratory flow between 25% and 75% of expiration; FEV_0.5_, forced expiratory volume during the first half-second of expiration; ND, not done; RV, residual volume; TLC, total lung capacity; % pre, percent predicted.

**Fig 2:**
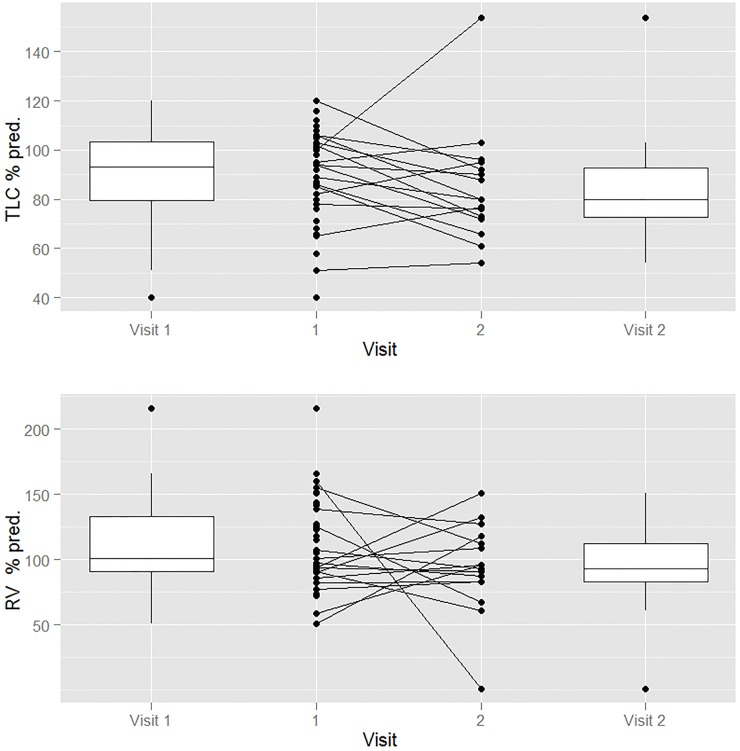
The residual volume (RV) and total lung capacity (TLC) in percentage of predicted volumes for the first and second pulmonary function tests as boxplots of the median and interquartile ranges with lines between indicating the individual changes.

Thirty-six patients had plethysmography at the first visit and the results were abnormal (decreased TLC percent of predicted, elevated RV/TLC percent of predicted) in 18 patients. Fig 2 displays the RV and TLC as percentage of predicted for the patients for the first and second PFTs as boxplots of the median and interquartile ranges with lines between indicating the individual changes. Of the 17 patients who had a second plethysmography, six were and remained normal, eight were and remained abnormal, and three were normal and became abnormal (Tables 3 and 4).

Thirty-three patients were tested for bronchodilator responsiveness (improvement in one or more of the following: FEV_0.5_ 4.3 ± 4.0%, FEF_25–75%_ 11.2 ± 8.2%, FEF_75%_ 38.7 ± 5.3%, FEF_85%_ 44.1 ± 11.1%) [6]. Eighteen of the 33 did not respond. At the follow-up test 13 patients were tested (Tables 3 and 4). Five were and remained responders, two were and remained non-responders, three were non-responders and became responders, and three were responders and became non-responders.

Of the 20 patients with mild airway compromise (related PAS 1–2), 16 had abnormal initial PFTs. Two had normal values on the repeat PFT 6 months later. Of the 15 patients with moderate airway compromise (related PAS 3–5), eight had abnormal initial PFTs. One of the patients who had a normal initial PFT had an abnormal PFT on repeat testing 6 months later. Of the three patients who had severe airway compromise (related PAS 6–8), two had abnormal PFTs on both initial and repeat testing. The dysphagia severity and the PAS were tested with the binary outcome (normal-abnormal) to see if they correlated with the results of the spirometry and plethysmography using the chi square exact test. The dysphagia severity did not correlate with the spirometry results (*p* = 0.37) or the plethysmography results (*p* = 0.09). The PAS correlated with the spirometry results (*p* = 0.03) but not the plethysmography results (*p* = 0.36).

The relationship of normal and abnormal PFT results with the presence or absence of GER, normal or abnormal chest radiographs, passive smoke exposure or daycare exposure are summarized in Table 5. None of the variables correlated with abnormal PFTs.

**Table 5:** Correlation of pulmonary function test results in study subjects with gastroesophageal reflux disease, chest radiograph findings, and exposure to passive smoke or daycare.

Variables	Pulmonary Function Test	
	Abnormal	Normal
Gastroesophageal reflux (GER), n = 23/38	15	8
No GER, n = 15/38	13	2
Abnormal chest radiograph, n = 16/33	11	5
Normal chest radiograph, n = 17/33	15	2
Passive smoke exposure, n = 13/38	12	1
No passive smoke exposure, n = 25	16	9
Daycare exposure, n = 18/38	13	5
No daycare exposure, n = 20/38	17	3

## Discussion

There are very few published studies available about the use of PFTs in infants who have recurrent respiratory symptoms that could be caused by potential aspiration. No previously reported results of PFTs performed in infants with swallowing dysfunction are available. In our prospective observational pilot study, we demonstrated that 25 of the 38 infants diagnosed with swallowing dysfunction had abnormal spirometry and 18 had abnormal lung volumes.

Since 1991, three studies have reported on PFTs in infants with GER [11–13]. In those studies, none of the infants had documented swallowing dysfunction, no mention was made of aspiration in those infants, and PFTs were performed at tidal volume. In our study, PFTs were performed by RTC from total lung capacity and progressing to residual volume, giving more complete measurements than infant PFTs performed at TV.

There were potential confounding factors that occurred in our study. Twenty-three of the infants had both swallowing dysfunction and GER. We were unable to determine which of these problems, if not both, resulted in the infants’ respiratory symptoms and abnormal PFTs. Thirteen of the infants were exposed to tobacco smoke in their homes and 18 of them spent time in daycare centers. Thus, the infants could be wheezing and coughing due to exposure to the air pollution in their homes and/or to the respiratory viruses they encountered in the daycare centers. These factors could affect their infant PFT results. Thirty-four of the infants had a family history of asthma or atopy, which is associated with the development of recurrent wheezing in children. Because all of these confounding factors are so common in the general population, it would have been extremely difficult to enroll a sufficient number of study infants with swallowing dysfunction who had none of these confounding factors.

There are several possible reasons why the PFTs in the infants we studied did not significantly improve after receiving therapies for swallowing dysfunction and GER. First, the number of subjects was small, with only 17 of the original 38 infants returning to have second PFTs performed. Because of this, the power of the study was insufficient to show changes that were seen between the first and second PFTs to achieve statistical significance. Second, it may be that therapies for swallowing dysfunction/GER longer than 6 months, e.g., 1 year, are needed to achieve improvement in PFTs, and, unfortunately, some of the infants would have grown too large for infant PFTs. Third, compliance with therapies was assessed only by the parent(s)/caregiver(s) report. Noncompliance rates with swallowing dysfunction therapies in both adults and children are high [18,19]. There are no published data on compliance in infant populations. The noncompliance rate in our patient group is likely higher than what the families reported.

The severity of the patients’ swallowing dysfunction and aspiration may have been underestimated by the VFS for some of the patients. VFS is the “assumed” gold standard for diagnosing swallowing dysfunction in adults [20]. The sensitivity and specificity for detecting aspiration are reported to be 100% and 63%, respectively, in adults [20–23], but these parameters have not been reported in children. The same may be true for the patients’ GER and their therapies. GER was diagnosed in these infants using a barium esophagogram or by gastric scintiscan. These tests are not as sensitive as a 24-hour pH probe study in determining the frequency and severity of GER in these infants. In patients who had both swallowing dysfunction and GER, it was impossible to discriminate which process, if not both, was associated with their abnormal lung function.

Finally, it may be that patients had a primary lung process detected by PFTs that was not due to swallowing dysfunction or aspiration. We excluded patients with known lung diseases, but it is possible that the infants already had mild structural changes, such as early bronchiectasis, that might not improve over time. Lung computed tomography imaging might be more sensitive to these mild structural changes.

In this study, it was necessary to use published normal reference values for infant PFTs and for assessment of bronchodilator responsiveness [6,7]. Use of such equations may lead to misinterpretation of lung function status [24], which could have adverse effects in both the research setting and on clinical management. Lum recommended healthy control infants be recruited for studies involving infant PFTs [24], and several groups have done so [25–31]. There is currently debate as to whether it is ethical to perform PFTs under sedation in healthy infants. Many institutional review boards, including our own, feel that the risks of sedating normal infants for PFTs outweighs the potential benefits to them. The technique and equipment for infant lung function testing are now well standardized and we believe the use of published normal values is now practical.

Despite these many limitations, we feel that it is still valuable that we have shown that infants with swallowing dysfunction frequently have abnormal infant PFTs. We were able to characterize the type of lung PFT abnormality as obstructive, restrictive, or both, as well as the severity of that abnormality. An abnormal PFT could not be predicted by the severity of a patient’s airway compromise during the swallow (as rated by PAS), the presence or absence of GER, chest radiograph abnormalities, or attendance in daycare. The majority of patients who were exposed to tobacco smoke had abnormal PFT results. We did not see a significant change in PFT findings in the majority of the patients in the short term using accepted therapies for swallowing dysfunction/GER. Future prospective studies are needed to determine if longer courses of therapy will improve/correct these PFT abnormalities, and whether these PFT abnormalities persist into school-age.
